# Treatment with Docosahexaenoic Acid Improves Epidermal Keratinocyte Differentiation and Ameliorates Inflammation in Human Keratinocytes and Reconstructed Human Epidermis Models

**DOI:** 10.3390/molecules24173156

**Published:** 2019-08-30

**Authors:** Tinghan Jia, Wu Qiao, Qifeng Yao, Wenhui Wu, Ken Kaku

**Affiliations:** 1 Pigeon Maternal & Infant Skin Care Research Institute, Shanghai 201700, China; 2 Department of Marine Bio-Pharmacology, College of Food Science and Technology, Shanghai Ocean University, Shanghai 201306, China

**Keywords:** docosahexaenoic acid, reconstructed human models, filaggrin, skin barrier, inflammation

## Abstract

Atopic dermatitis (AD) is a chronic inflammatory skin disease that can cause skin barrier function damage. Although co-incubation with docosahexaenoic acid (DHA) exerts a positive effect on deficient skin models, no studies have investigated the effects of topical treatment with DHA in an inflammatory reconstructed human epidermis (RHE) model. The effects of DHA on monolayer normal human epidermal keratinocyte (NHEK) cells were evaluated using cell counting kit-8 (CCK-8), real-time quantitative polymerase chain reaction (qPCR), and enzyme-linked immunosorbent assay (ELISA). The skin-related barrier function was assessed using hematoxylin–eosin (HE) staining, Western blot (WB), immunohistofluorescence (IF), and ELISA in normal and inflammatory RHE models. Docosahexaenoic acid upregulated filaggrin and loricrin expression at mRNA levels in addition to suppressing overexpression of tumor necrosis factor-α (TNF-α), interleukin-α (IL-1α), and interleukin-6 (IL-6) stimulated by polyinosinic–polycytidylic acid (poly I:C) plus lipopolysaccharide (LPS) (stimulation cocktail) in cultured NHEK cells. After topical treatment with DHA, cocktail-induced inflammatory characteristics of skin diseases, including barrier morphology, differentiation proteins, and thymic stromal lymphopoietin (TSLP) secretion, were alleviated in RHE models. Supplementation with DHA can improve related barrier function and have anti-inflammation effects in monolayer keratinocytes and RHE models, which indicates that DHA may have potential value for the treatment of inflammation-associated skin diseases.

## 1. Introduction

The skin barrier, consisting of dermis and upper epidermis, is the first physical protective barrier of the human body. The epidermis, a multi-layered compartment, protects humans from negative external environmental factors by preventing foreign pathogens, reducing water loss, and maintaining the homeostasis of skin through cell death and differentiation of keratinocyte [1,2]. Filaggrin originates from its larger precursor profilaggrin, which exists in keratohyalin granules and is located in the stratum granulosum. During terminal differentiation, profilaggrin generates filaggrin monomers that can aggregate and link keratin to form a cornified envelope (CE), which has a critical role in maintaining the integrity of the stratum corneum (SC) [3]. Meanwhile, filaggrin is a core epidermal protein that plays an important role for its intracellular metabolites that make contributions to epidermal barrier functions such as SC hydration and inhibition of UV-B irradiation [4].

Some common skin barrier diseases, such as atopic dermatitis (AD) and ichthyosis vulgaris (IV), are connected with a filaggrin deficiency like filaggrin gene mutations [5]. It has been identified that loss-of-function filaggrin (FLG) is the most important genetic risk factor for AD patients and is a potential pathogenic factor for IV [6]. However, variation of FLG genes is not the only factor that causes filaggrin deficiency; the lack of filaggrin can also be observed in AD patients without an FLG mutation status [7]. More and more studies have found the modulatory effects of an inflammatory environment in the epidermis. One of the major symptoms of AD patients is the overexpression of interleukin-4 (IL-4) and interleukin-13 (IL-13), which clearly decreases the filaggrin expression in normal human epidermal keratinocyte (NHEK) cells [8,9]. The dysregulation or the absence of filaggrin can cause skin barrier dysfunction, aggravate inflammation, and increase the risk of microbial infections [10].

Traditionally, many studies have proposed effective programs, including alleviating Th2-mediated inflammation and immunosuppressive drugs, to treat skin diseases in AD patients. However, recent studies have shown that these traditional therapies can cause short- or long-term risks [11,12]. There is increasing evidence that filaggrin deficiency is at the core of AD pathogenesis; hence, upregulation of filaggrin expression is a more logical, effective, and safe barrier repair therapy. Peroxisome proliferator-activated receptor (PPAR) agonists, especially PPARα and PPARγ, have drawn attention to the treatment of filaggrin-associated diseases, including skin homeostasis and anti-inflammation, which allow PPAR agonists to be further explored [13,14]. However, most of the results of topical treatment are from mouse models, which have interspecific differences [15]. Even if recent evidence shows that PPARα + γ agonist docosahexaenoic acid (DHA) can upregulate the expression of FLG in organotypic, normal, and deficient reconstructed human epidermis (RHE) skin models, all models are incubating with DHA [16,17]. More importantly, there is almost no research on whether the DHA works in inflammatory reconstructed human skin.

In this work, we first investigated the effects of DHA in the human keratinocyte monolayer. Second, we investigated the impacts of a topical treatment with a simple DHA formula in normal and inflammatory RHE skin models for the first time. We follow with interest the positive effects of DHA on the structural protein expression, skin barrier function, and anti-inflammation, aiming for novel insights into treatment with DHA in related skin diseases.

## 2. Results

### 2.1. Effect of DHA, Polyinosinic–Polycytidylic Acid (poly I:C) Plus Lipopolysaccharide (LPS) on Cell Viability

**Figure 1:**
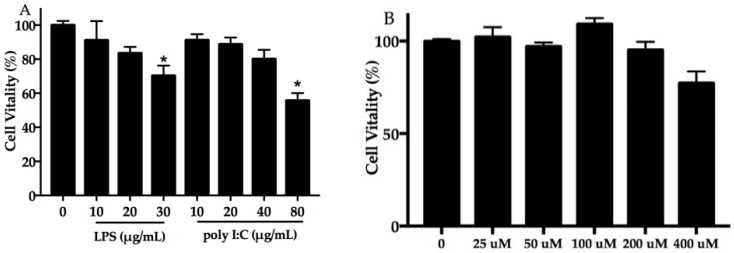
Effect of poly I:C, LPS, and docosahexaenoic acid (DHA) on cell viability. Normal human epidermal keratinocyte (NHEK) cells exposed to poly I:C (0–80 μg/mL) and LPS (0–30 μg/mL) (**A**), and DHA (**B**) for 24 h. Data are expressed as mean ± standard deviation (SD), *n* = 5. * Compared with blank control, *p* < 0.05. LPS, lipopolysaccharide; poly I:C, polyinosinic–polycytidylic acid.

The viability of NHEK cells was assessed using a cell counting kit-8 (CCK-8) assay. The results presented in Figure 1A show that NHEK cell viability decreased with the increasing concentration of poly I:C and LPS. Compared with the blank control group (*p* < 0.05), 30 μg/mL LPS and 80 μg/mL poly I:C significantly suppressed NHEK cell viability. As shown in Figure 1B, 200 μM of DHA had no apparent effect on cell proliferation. It is interesting that cell proliferation was higher in the 100-μM DHA supplement group than the control group, which was not obviously statistically significant.

### 2.2. DHA Decreased the Cocktail-Stimulated Proinflammatory Genes and Cytokine Secretion in NHEK Cells

**Figure 2:**
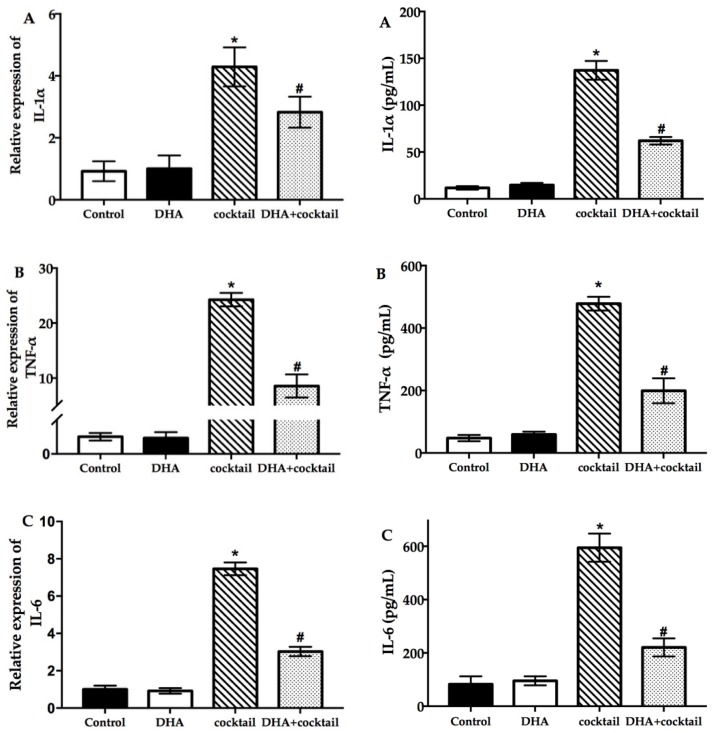
Effect of DHA on the inflammatory cytokine expression in NHEK cells with or without the cocktail (20 μg/mL LPS plus 10 μg/mL poly I:C). The results of real-time quantitative polymerase chain reaction and enzyme-linked immunosorbent assay show the changes of IL-1α (**A**), TNF-α (**B**), and IL-6 (**C**) after incubation for 24 h. Data are presented as mean ± SD, *n* = 5. * Compared with the control group, *p* < 0.05; # compared with the cocktail (LPS plus poly I:C) treated group, *p* < 0.05. IL, interleukin; TNF, tumor necrosis factor; cocktail, 20 μg/mL LPS plus 10 μg/mL poly I: C.

To evaluate the effect of DHA on the stimulation cocktail (the concentration of cocktail is shown in Figure S1) and spontaneous proinflammatory cytokine expression in NHEK cells, this study measured interleukin-1α (IL-1α) (Figure 2A), tumor necrosis factor-α (TNF-α) (Figure 2B), and interleukin-6 (IL-6) (Figure 2C) expression at the levels of mRNA and protein. As shown in Figure 2, the results of proinflammatory genes and secreted protein in the stimulation cocktail groups significantly (*p* < 0.05) increased compared to the control group. Interestingly, the addition of DHA significantly decreased the proinflammatory gene and cytokine expression induced by the cocktail (Figure 2) (*p* < 0.05).

### 2.3. Effect of DHA on Cultured NHEK Cells with or without Stimulation Cocktail

**Figure 3:**
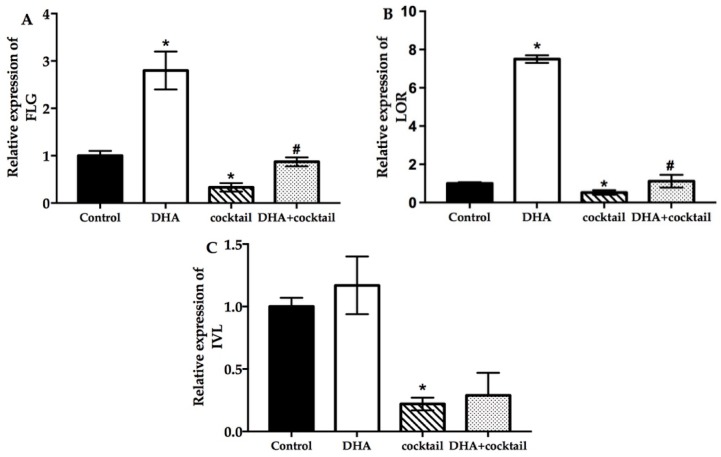
Effect of DHA on the differentiation of cultured NHEK cells with or without cocktail (20 μg/mL LPS plus 10 μg/mL poly I: C. Real-time quantitative polymerase chain reaction was used to evaluate the changes in FLG (**A**), LOR (**B**), and IVL (**C**) mRNA expression after incubation for 24 h. Data are presented as mean ± SD, *n* = 5. * Compared with a respective blank group, *p* < 0.05; # compared with their respective cocktail (20 μg/mL LPS plus 10 μg/mL poly I:C)-alone-treated group, *p* < 0.05. FLG, filaggrin; LOR, loricrin; IVL, involucrin.

The relative marker gene filaggrin (FLG), loricrin (LOR), and involucrin (IVL) expression was assessed for analysis of the effect of the keratinocyte differentiation in response to DHA with or without stimulation cocktail. Incubation with DHA significantly increased the expression of FLG and LOR compared with the blank group (Figure 3). The FLG and LOR were upregulated 2.7-fold and 7.2-fold after treatment with DHA, respectively. Interestingly, the expression of IVL was marginally influenced by DHA (Figure 3). In contrast, the stimulation cocktail significantly inhibited the expression of FLG, IVL, and LOR. After the DHA supplement was added, the amount of FLG and LOR in the stimulation cocktail-treated group was 2.1-fold and 4.1-fold, respectively, which was higher than the stimulation-cocktail-alone-treated group. No difference was observed in the IVL.

### 2.4. Topical Treatment with DHA Stimulates Differentiation and Improves Barrier Homeostasis

**Figure 4:**
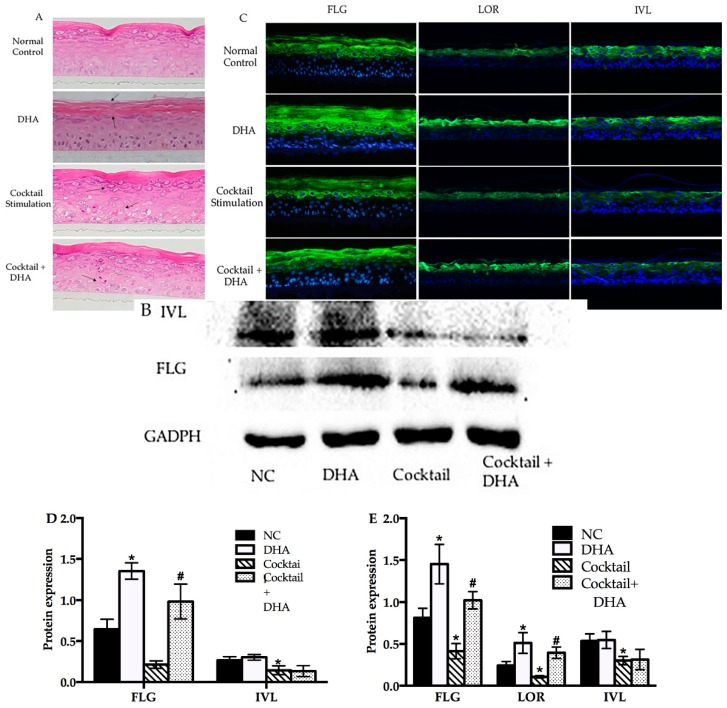
Topical treatment with DHA affected the morphology and differentiation protein expression in normal and inflammatory RHE models. (**A**) Morphology of normal, DHA cocktail-induced, and DHA-treated groups were evaluated using hematoxylin–eosin (HE) staining. (**B**) Western blot of FLG and IVL in normal control, DHA cocktail-induced, and DHA-treated groups. (**C**) Immunohistofluorescence analysis was executed for FLG, LOR, and IVL in the above RHE models. (**D**) Relative optical densities of FLG and IVL. (**E**) Relative fluorescence densities of FLG, IVL, and LOR. Magnification 10×, mean ± SD, *n* = 4–5. * Compared with the blank control groups, *p* < 0.05; # compared with the inflammatory group, *p* < 0.05. RHE, reconstructed human epidermis.

Docosahexaenoic was not sufficiently stable to attach on the RHE; hence, we made a simple formula (Table S2) to investigate the effect of topical treatment with DHA. First, we investigated the morphology of normal and inflammatory RHE models topically treated with DHA, which was done by using hematoxylin–eosin staining. As shown in Figure 4A, the stratum corneum thickness of RHE significantly increased after topical treatment with DHA compared with the normal group. Meanwhile, living layers of inflammatory RHE models induced by the stimulation cocktail were looser and inflammatory RHE had spongiosis compared with normal models. Interestingly, the barrier disruption of inflammatory RHE models significantly improved after topical treatment with the DHA formula. As shown in Figure 4A, there is a clear trend of decreasing cavities in living layers and the status of spongiosis remarkably improved. Next, the effects of DHA on RHE differentiation protein were analyzed using immunohistofluoresence and Western blot analysis. After topical treatment with DHA, the amount of FLG and LOR significantly increased compared with the normal group (Figure 4B,C). The results of image quantification of immunohistofluoresence and Western blot also revealed that FLG synthesis significantly improved (*p* < 0.05). However, there was no significant effect on IVL in DHA-treatment group (Figure 4), which was in agreement with the result of qPCR. The FLG, LOR, and IVL expression in inflammatory RHE induced by stimulation cocktail was significantly decreased compared with the normal groups, as shown in the results of WB and IF (Figure 4B,C). In inflammatory RHE models, topical treatment with DHA significantly improved the FLG and LOR expression. Again, DHA also showed no effect on the expression of IVL in inflammatory RHE (Figure 4).

### 2.5. Topical Treatment with DHA Downregulated the Release of Thymic Stromal Lymphopoietin (TSLP) in the Inflammatory RHE Model

**Figure 5:**
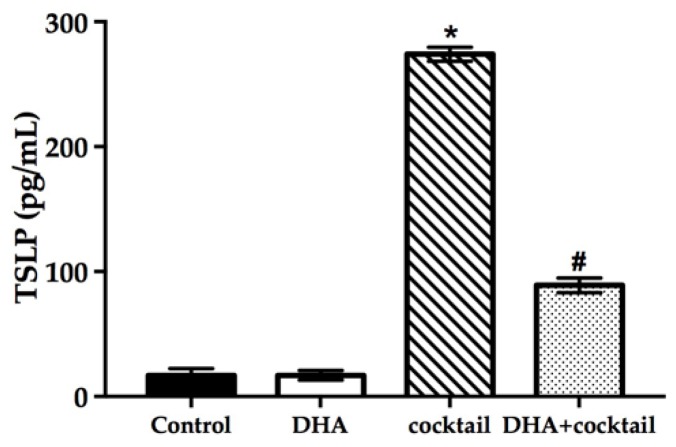
Effect of DHA on the TSLP secretion of RHE models with or without 20 μg/mL LPS plus 10 μg/mL poly I: C. The changes of TSLP were assessed using ELISA. Data are expressed as mean ± standard deviation (SD). * Compared with the normal control, *p* < 0.05; # compared with the inflammatory group, *p* < 0.05. TSLP, thymic stromal lymphopoietin.

It was necessary to prove that the inflammatory RHE model has a reference value, so we measured the level of TSLP using ELISA, which is related to the inflammation characteristics observed in AD patients. As shown in Figure 5, supplementation with the cocktail significantly increased the secretion of TSLP compared with normal RHE models (16 pg/mL vs. 274 pg/mL, *p* < 0.05). Docosahexaenoic acid alone showed a slight effect on TSLP expression in normal RHE models. On the contrary, the topical treatment with DHA significantly decreased TSLP expression in inflammatory RHE models (90 pg/mL vs. 270 pg/mL, *p* < 0.05).

## 3. Discussion

The defect of filaggrin is a primary pathogenic factor for AD [18]. Many studies have demonstrated that topical treatment with glucocorticoids and calcineurin inhibitor can decrease the integrity of the stratum corneum and destroy skin functions [12,19]. Therefore, the core of AD treatment is to repair skin barriers and restore relative function. The general approaches to cure AD comprise hydration, regulation of SC pH [20], and application of epidermal barrier-improving agents such as liver X receptor (LXR) activators, adenosine monophosphate (AMP)-increasing agents, and PPAR activators [21]. The PPARs, including PPARα, PPARβ/δ, and PPARγ, are expressed in human keratinocytes and skin, and play a critical role in keratinocyte differentiation and skin recovery [22]. All epidermal layers contain PPARβ/δ, but PPARα and PPARγ are present in the epidermal suprabasal layer. Qiang [23] and Yan [24] have demonstrated that PPARγ agonists can stimulate cultured human keratinocyte differentiation and repair the skin barrier in mouse models. Meanwhile, a PPAR-α agonist like WY14643 increases the expression of some epidermal differentiation structural proteins, which may be critical in human keratinocyte differentiation. Besides, PPAR activators can alleviate and remedy the adverse effects of topical glucocorticoids (GC), like decreased keratinocyte proliferation and differentiation in skin [25]. Docosahexaenoic acid can act as a dual PPARα/γ agonist [26], and in the current study, we investigated the impact of DHA in monolayer culture human keratinocytes. We also found that DHA significantly increased the expression of FLG and LOR in RNA levels in vitro, without any effect on IVL (Figure 2). However, monolayer culture human cells cannot present complete epidermal maturation characteristics. Several reports have shown that PPAR agonists can reverse damaged barrier function in atopic dermatitis-like model; for example, Chiba and Yoshida found that topical application of a PPAR-α agonist and DHA can treat AD in NC/Nga mouse models [27,28]. Some studies found that rodent skin models might be able to explain some skin reactions to PPARs, yet there are restrictions on species specificity and interspecific difference [29,30].

Furthermore, it has been demonstrated that there is a presence of PPARs in reconstructed skin. The expression of filaggrin and other functional proteins significantly increases with PPAR agonist supplement in normal models [31]. In the literature, it was found that DHA has difficulty effectively adhering to the skin; hence, all data were based on the association between incubation with DHA and normal or FLG-deficient models, whereas very little literature is based on the question of topical DHA application in reconstructed human epidermis (RHE) models. Only a small clinical trial indicated a therapeutic effect in AD patients after topical application for two weeks [32]. In the present study, we firstly generated a normal RHE to investigate the topical effects of DHA. Second, we made a simple formula (Table S2) to ensure that DHA can stably attach to the surface of RHE. We also observed that SC became thicker after topical treatment with DHA (Figure 3), which agrees with previous findings [31]. Another important function of filaggrin is its dephosphorylation degradation product, natural moisturizing factor (NMF), which affects multiple crucial functions in the maintenance of epidermal homeostasis. Natural moisturizing factor not only modulates the skin pH and acidification and increases water retention, but also shows the inhibitory influence of pathogenic microorganism colonization and has a positive effect on filaggrin-processing enzyme activity [33]. Thus, we demonstrated for the first time that upregulation of filaggrin works in RHE models after topical treatment with a DHA formula and DHA also increases filaggrin and loricrin expression. Interestingly, IVL was not affected by DHA (Figure 3).

As mentioned in the literature review, an inflammatory environment and microbial infection are other important risk factors for inducing and aggravating AD. Lipopolysaccharide, a pathogen-associated molecular pattern (PAMP), can trigger toll-like receptors (TLRs) to produce pro-cytokines in human keratinocytes [34], which can act as a microbial infection to generate an inflammatory environment in this model. Poly I:C is known as a TLR3 ligand stimulator that can imitate double-stranded RNA to induce an acute immune response in human keratinocytes. Although there are some AD-like feature RHE models induced by certain regulatory factors in previous studies [35], this study first developed an AD-associated feature RHE model induced using poly I:C plus LPS. The concentrations of poly I:C and LPS were chosen according to Figure S1 and previous research [36,37]. After treatment of RHE with the stimulation cocktail, the epidermal morphology was changed and brought about spongiosis, which agrees with other AD-like models [38] and the characteristics of AD patients [39] (Figure 4). It is interesting to note that the results of HE are well in line with morphological characteristics of epidermal models induced by IL-3 and IL-14 [40]. Meanwhile, the stimulation cocktail decreased the distribution of barrier proteins like FLG, LOR, and IVL (Figure 5), which is similar to other AD-like skin models in vitro [41,42]. Here, we first demonstrated that the upregulation of FLG and LOR works in inflammatory RHE models after supplementary topical treatment with DHA (Figure 4).

Thymic stromal lymphopoietin that was abundantly expressed by keratinocytes plays an important part in AD and other allergic disorders [43]. Prior studies have noted that TSLP is governed by the nuclear factor kappa-B (NF-κB) pathway through TLR3. Thymic stromal lymphopoietin is over-expressed in RHE models induced by a stimulation cocktail, which is associated inversely with FLG expression in AD patients. More interestingly, high-level proinflammatory cytokines (IL-1α and TNF-α) can induce TSLP expression in keratinocytes [44]. Simultaneously, TSLP can cause a Th2 inflammatory reaction via a vicious circle, which is a potential underlying pathogenesis of AD and the atopic march [36]. In this study, the cocktail can significantly stimulate the proinflammatory cytokines secretion in monolayer culture human keratinocytes (Figure 2). The cytokine levels of IL-1α were correlated inversely with NMF levels. Conversely, the levels of cytokines and TSLP secretion were lower than the DHA-untreated group. It is possible, therefore, that DHA can increase FLG expression by decreasing the expression of TSLP and proinflammatory cytokines in inflammatory RHE models.

## 4. Materials and Methods

### 4.1. Monolayer Cell Culture

Normal human epidermal keratinocyte cells were purchased from Guangdong Biocell Co., Ltd. (Guangdong, China) and cultured in EpiLife (Gibco, Thermo Fisher Scientific, Waltham, MA, USA) medium containing 60 μM Ca^2+^ and HKGS (Gibco, Thermo Fisher Scientific) in a 5% CO_2_ incubator (Thermo Fisher Scientific) at 37 °C. The medium was replaced every two days, and the cells were used at 70% to 80% confluence.

### 4.2. Cell Viability Assay

Cell Counting Kit-8 (Beyotime Biotechnology, Shanghai, China) was used to test NHEK cell viability with a high accuracy. Normal human epidermal keratinocyte cells (6 × 10^3^ cells/well) were seeded in 96-well plates (Nunc, Thermo Fisher Scientific). After being stimulated with different concentrations of poly I:C, LPS, and DHA for 24 h, the NHEK cells were incubated at 37 °C for 4 h, adding 10 μL/100 μL CCK-8. The results of optical density (OD) was measured by reading the absorbance at 450 nm with a Microplate Reader (Molecular Devices, San Francisco, CA, USA). The cell viability ratio was calculated, and the calculation formula is as follows:

$$\mathrm{Viability}\;\left(\%\right)\;=\;\left(\frac{\mathrm{OD}\;\mathrm{treatment}\;\mathrm{group}}{\mathrm{OD}\;\mathrm{blank}\;\mathrm{control}\;\mathrm{group}}\right)\;\times \;100$$

### 4.3. Study Design and DHA Supplement

For LPS and poly I:C, we determined the optimum concentrations for subsequent studies. Normal human epidermal keratinocyte cells (6 × 10^3^ cells/well) were seeded in 96-well plates (Nunc, Thermo Fisher Scientific) and treated with different concentrations of poly I:C and LPS for 24 h according to the results of cell viability. We defined the best concentration of LPS plus poly I:C by measuring the expression of TSLP (Figure S1).

Normal human epidermal keratinocyte cells were seeded into six-well plates at a density of 3 × 10^4^ cells/mL. The NHEK cells were cultured in EpiLife medium with 1.3–1.5 μM Ca^2+^ and HKGS for 72 h, and the medium was replaced with fresh EpiLife medium containing a different DHA (100 μM) for an extra 24 h. For the inflammatory stimulation, NHEK cells were incubated with HKGS and 1.5 μM Ca^2+^ in EpiLife medium for 72 h. After the cocktail was added to the medium to stimulate for 24 h, 100 μM DHA was added to the medium for another 24 h.

### 4.4. Quantitative Real-Time PCR Analysis

The total RNA was extracted from the NHEK cells following the TRIZOL reagent (Life Technologies, Carlsbad, CA, USA) recommended protocol. The concentration and quality of RNA were measured using Qubit 3.0 (Thermo Fisher Scientific). This RNA was used for subsequent cDNA synthesis with the PrimeScript RT reagent Kit (Thermo Fisher Scientific). The changes in mRNA levels were measured using a Light Cycler 96 system (Roche) and SYBR Premix Ex Taq II (Takara Biotechnology, Dalian, China) following the manufacturer’s recommended protocol. All primers of FLG, LOR, IVL, IL-1α, TNF-α, IL-6, and glyceraldehyde-3-phosphate dehydrogenase (GADPH) are listed in Table S1. The related data were analyzed using the delta cycle threshold method and the relative expression levels of each gene were normalized to the Ct of the glyceraldehyde 3-phosphate dehydrogenase and calculated based on the 2^−ΔΔCT^ method.

### 4.5. Construction of RHE Models and DHA Topical Treatment

The RHE model was established using a complete EpiLife growth medium and Cell Culture Inserts (Thermo Fisher Scientific). Briefly, complete EpiLife growth medium was prepared by adding 10 ng/mL keratinocyte growth factor, human keratinocyte growth supplement, 50 μg/mL ascorbic acid, and 140 μM CaCl_2_. According to the standard protocol, a coating matrix was diluted by 1:100 and precoated. Cells were seeded in precoated inserts with a 0.5 mL growth medium in both lower and upper compartments at a density of 7.5 × 10^4^ cells/cm^2^. The air–liquid interface was established by adding complete growth medium with additional 1.5 mM CaCl_2_ to the lower compartment and aspirating the upper compartment medium for 2 days’ incubation at 37 °C and 5% CO_2_. Changes to the subsequent medium were made by aspirating the medium from the lower compartment and replacing it with fresh medium supplemented with 1.5 mM CaCl_2_ (1.7 mM total CaCl_2_). For inflammatory RHE, the cocktail was added 72 h before the DHA treatment.

According to Table S2, a simple formula was designed and used for the next study. We dispensed 32 μL/cm^2^ of the 0.1% DHA cream formula on the top of each epidermis tissue. After 24 h of incubation, it was rinsed thoroughly 25 times with 1 mL PBS to remove all residual chemicals from the epidermal surface before further analysis.

### 4.6. Haematoxylin–Eosin Staining

Harvested RHE models were fixed in 10% formalin reagent (Sigma, St. Louis, MO, USA), dehydrated with ethanol, and embedded in paraffin. Sections of RHE were cut to 5 μm, deparaffinized, and rehydrated using a graded ethanol series. Finally, the slices were stained with hematoxylin and eosin (Sigma). The RHE slices were observed using EVOS FL Auto (Thermo Fisher Scientific) after mounting with a neutral balsam.

### 4.7. Western Blot

After treatment, the RHE models were washed with cold PBS and harvested with M-PER Mammalian Protein Extraction Reagent (Thermo Fisher Scientific). A bicinchoninic Protein Assay kit (Thermo Fisher Scientific) was used to measure the protein concentrations. Then, 20 μg proteins were boiled and separated using 10% sodium dodecyl sulfate polyacrylamide gel electrophoresis (SDS-PAGE), and gels were transferred onto a polyvinylidene fluoride (PVDF) membrane by using a Power Blotter System (Thermo Fisher Scientific). Following 5% skimmed-milk blocking, the membranes were incubated with anti-FLG (1:2000; Thermo Fisher Scientific), anti-IVL (1:2000, Novus Biological, Bio-Techne, Minneapolis, MN, USA), and anti-GAPDH (1:5000; Invitrogen, Thermo Fisher Scientific) antibodies overnight at 4 °C. The membranes were washed and incubated with a secondary antibody (1:20,000) (Invitrogen, Thermo Fisher Scientific) conjugated with horseradish peroxidase (HRP) in 0.5% PBST for 1 h. Protein expression was detected using an iBright FL1000 image system (Thermo Fisher Scientific) and quantified using iBright analysis software 3.0.0 (Thermo Fisher Scientific).

### 4.8. Immunohistofluorescence Assay

After topical treatment, the RHE models were washed with cooled PBS and fixed with 4% paraformaldehyde for 30 min. After incubation in 0.05% Triton X-100 for 30 min, the treated RHE slices were blocked with a 5% bovine serum albumin (BSA) buffer for 60 min. The RHE models were incubated with primary antibody (FLG 1:500, LOR 1:500, IVL 1:500, Abcam, Cambridge, UK) overnight at 4 °C, followed by incubation with an Alexa Fluor 488-conjugated secondary goat anti-rabbit IgG (1:200; Invitrogen) for 2 h in the dark at 37 °C. The nuclei were stained using incubation for 10 min at room temperature with 4,6-diamidino-2-phenylindole (DAPI, 1:1000, Thermo Fisher Scientific). The images of immunohistofluorescence were visualized with a fluorescence microscope (EVOS FL Auto, Life Technology, Carlsbad, CA, USA) and analyzed using the EVOS browser imaging software 1.7 (Thermo Fisher Scientific).

### 4.9. TNF-α, TSLP IL-α, and IL-6 Using ELISA

The supernatant of NHEK cells or RHE models’ samples were collected from the six-well plates. Proinflammatory mediators like TNF-α, IL-1α, IL-6, and TSLP were determined using ELISA kits (Bio-Techne). The tests were performed strictly according to the manufacturer’s instructions.

### 4.10. Statistical Analyses

All the values have been reported in terms of means ± SD. The mean values were calculated based on data from at least three independent replicate experiments. The data were analyzed using Student’s *t*-test. A *p*-value of less than 0.05 was statistically significant. All statistical analyses were performed using SPSS 25.0 (IBM Co., Armonk, NY, USA)

## 5. Conclusions

In conclusion, treatment with DHA can improve related barrier function and ameliorate inflammation in monolayer keratinocytes and inflammatory RHE models, which indicates that DHA may have potential value for the treatment of inflammatory-associated skin diseases.

## Supplementary Materials

The following are available online, Figure S1: Effect of poly I:C, LPS, and a cocktail on TSLP secretion in NHEK cells. Table S1: Primers for qPCR. Table S2: Formula composition.
