# Supplementary material for: Treatment with Docosahexaenoic Acid Improves Epidermal Keratinocyte Differentiation and Ameliorates Inflammation in Human Keratinocytes and Reconstructed Human Epidermis Models
Source: Molecules. 2019 Aug 30;24(17):3156. doi: 10.3390/molecules24173156 (PMC6749566; doi:10.3390/molecules24173156)
Supplement: Supplementary file 1 [file molecules-24-03156-s001.pdf]

Table S1. Primers for qPCR

Primer	Sequences (5′–3′)	Gene Bank ID
IL6 F	ACTCACCTCTTCAGAACGAATTG	NM_000600.5
IL6 R	CCATCTTTGGAAGGTTCAGGTTG	
IL-1α F	ATGGCCAAAGTTCGAGACATG	NM_000575.4
IL-1α R	CTACGCCTGGTTTTCCAGTATCTGA	
TNFα F	ATGAGCACTGAAAGCATGATCC	NM_000594.4
TNFα R	GAGGGCTGATTAGAGAGAGGTC	
FLG F	GCAAGGTCAAGTCCAGGAGA	NM_00206.1
FLG R	CCCTCGGTTTCCACTGTCTC	
LOR F	GAGTTGGAGGTGTTTTCCAGGG	NM_000427.3
LOR R	GCAGAACTAGATGCAGCCGGA	
IVL F	TAACCACCCGCAGTGTCCAG	NM_005547.3
IVL R	ACAGATGAGACGGGCCACCTA	
GADPH F	GAAGGTCGGAGTCAACGGATT	NM_002046.7
GADPH R	TGACGGTGCCATGGAATTTG	

Table S2. Formula Composition

Ingredient	Content (%)
Carbomer	1%
Propylene glycol	15%
Water	100%
DHA	0.1%

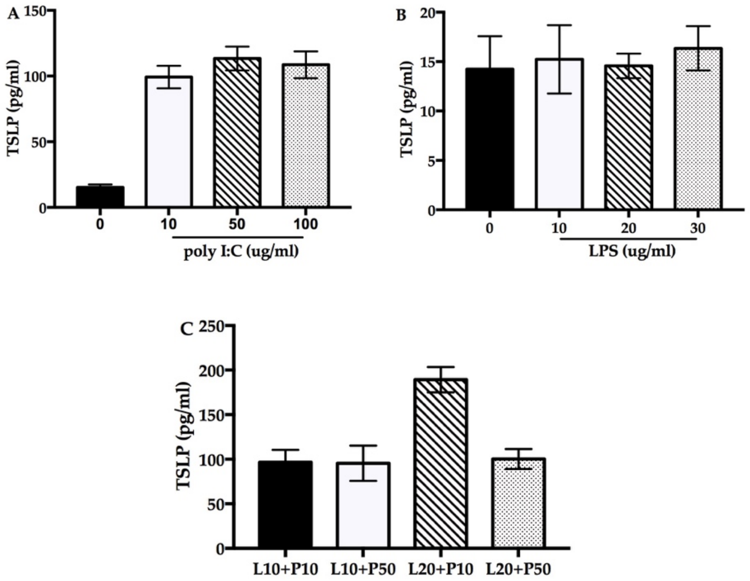

Figure S1. Effect of poly I:C, LPS, and a cocktail on the TSLP secretion in NHEK cells. The changes of TSLP were accessed using ELISA. (**A**) Effects of poly I:C on the TSLP secretion. (**B**) Effects of LPS on the TSLP secretion. (**C**) Effects of LPS + poly I:C on the TSLP secretion. Data are expressed as mean ± standard deviation (SD). Cocktail, LPS + poly I:C; L, LPS, lipopolysaccharide; P, poly I:C, polyinosinic-polycytidylic acid; TSLP, thymic stromal lymphopoietin.

Methods

Regarding LPS and poly I:C, we determined the optimum concentrations for subsequent studies. NHEK cells (6 × 10^3 cells/well) were seeded in 96-well plates (Nunc, Thermo Fisher Scientific, Waltham, MA, USA) and treated with different concentrations of poly I:C, LPS, and poly I:C + LPS for 24 h according to the results of cells viability. We defined the best concentration of LPS plus poly I:C by measuring the expression of TSLP. The supernatant of NHEK cells were collected from the six-well plates. Thymic stromal lymphopoietin (TSLP) were determined by using ELISA kits (R&D System Inc., Minneapolis, MN, USA). The tests were performed strictly according to the manufacturer's instructions.

Results

Finally, we combined 20 μg/ml LPS and 10 μg/ml poly I:C for 24 hours and found that the expression of TSLP in the cocktail was significantly higher than another mixed group.
